# Terminally differentiated cytotoxic CD4 ^+^ T cells were clonally expanded in the brain lesion of radiation‐induced brain injury

**DOI:** 10.1111/cns.14682

**Published:** 2024-03-18

**Authors:** Xueying Ma, You Zuo, Xia Hu, Sitai Chen, Ke Zhong, Ruiqi Xue, Shushu Gui, Kejia Liu, Shaojian Li, Xiaoqiu Zhu, Jingwen Yang, Zhenhong Deng, Xiaolu Liu, Yongteng Xu, Sheng Liu, Zhongshan Shi, Meijuan Zhou, Yamei Tang

**Affiliations:** ^1^ Department of Neurology, Sun Yat‐sen Memorial Hospital Sun Yat‐sen University Guangzhou China; ^2^ Brain Research Center, Sun Yat‐sen Memorial Hospital Sun Yat‐sen University Guangzhou China; ^3^ Department of Radiation Medicine, Guangdong Provincial Key Laboratory of Tropical Disease Research, School of Public Health Southern Medical University Guangzhou China; ^4^ Jiangmen Central Hospital Affiliated Jiangmen Hospital of Sun Yat‐sen University Jiangmen China; ^5^ Department of Pharmacy, Sun Yat‐sen Memorial Hospital Sun Yat‐sen University Guangzhou China; ^6^ Department of Anesthesiology, Sun Yat‐sen Memorial Hospital Sun Yat‐sen University Guangzhou China; ^7^ State Key Laboratory of Ophthalmology, Zhongshan Ophthalmic Center, Guangdong Provincial Key Laboratory of Ophthalmology and Visual Science Sun Yat‐sen University Guangzhou China

**Keywords:** adaptive immunity, CD4^+^ CTL, clonal expansion, radiation‐induced brain injury, TCR sequencing

## Abstract

**Background:**

Accumulating evidence supports the involvement of adaptive immunity in the development of radiation‐induced brain injury (RIBI). Our previous work has emphasized the cytotoxic function of CD8^+^ T cells in RIBI. In this study, we aimed to investigate the presence and potential roles of cytotoxic CD4^+^ T cells (CD4^+^ CTLs) in RIBI to gain a more comprehensive understanding of adaptive immunity in this context.

**Main Text:**

Utilizing single‐cell RNA sequencing (scRNA‐seq), we analyzed 3934 CD4^+^ T cells from the brain lesions of four RIBI patients and identified six subclusters within this population. A notable subset, the cytotoxic CD4^+^ T cells (CD4^+^ CTLs), was marked with high expression of cytotoxicity‐related genes (*NKG7*, *GZMH*, *GNLY*, *FGFBP2*, and *GZMB*) and several chemokine and chemokine receptors (*CCL5*, *CX3CR1*, and *CCL4L2*). Through in‐depth pseudotime analysis, which simulates the development of CD4^+^ T cells, we observed that the CD4^+^ CTLs exhibited signatures of terminal differentiation. Their functions were enriched in protein serine/threonine kinase activity, GTPase regulator activity, phosphoprotein phosphatase activity, and cysteine‐type endopeptidase activity involved in the apoptotic signaling pathway. Correspondingly, mice subjected to gamma knife irradiation on the brain showed a time‐dependent infiltration of CD4^+^ T cells, an increase of MHCII^+^ cells, and the existence of CD4^+^ CTLs in lesions, along with an elevation of apoptotic‐related proteins. Finally, and most crucially, single‐cell T‐cell receptor sequencing (scTCR‐seq) analysis at the patient level determined a large clonal expansion of CD4^+^ CTLs in lesion tissues of RIBI. Transcriptional factor‐encoding genes *TBX21*, *RORB*, and *EOMES* showed positive correlations with the cytotoxic functions of CD4^+^ T cells, suggesting their potential to distinguish RIBI‐related CD4^+^ CTLs from other subsets.

**Conclusion:**

The present study enriches the understanding of the transcriptional landscape of adaptive immune cells in RIBI patients. It provides the first description of a clonally expanded CD4^+^ CTL subset in RIBI lesions, which may illuminate new mechanisms in the development of RIBI and offer potential biomarkers or therapeutic targets for the disease.

## Graphical abstract

A special CD4^+^ CTL subset is clonally expanded in the CNS lesion of RIBI, exhibiting a terminal differentiation state and enriched functions related to apoptotic signaling pathways. Transcriptional factors *TBX21, RORB,* and *EOMES* are positively correlated with the cytotoxic fucntions of CD4^+^ CTLs, offering potential markers to distinguish RIBI‐related CD4^+^ CTLs.

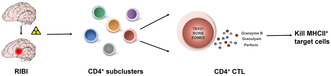

## 1 INTRODUCTION

Radiation‐induced brain injury (RIBI) is a severe complication in patients with head and neck tumors after radiotherapy, characterized by cognitive impairment, anxiety, headache, loss of appetite, and so on as its main symptoms.^1^, ^2^, ^3^ It dramatically affects the life quality of more than 50% of patients who undergo radiotherapy and survive.^4^ Clinically, glucocorticoids and bevacizumab are used as first‐line medicines,^5^, ^6^ and thalidomide and apatinib have passed phase II clinical trials according to previous researches.^7^, ^8^ However, current therapeutic approaches fall short of satisfaction due to a lack of knowledge regarding the pathogenesis. Although accumulating evidence has involved DNA damage, neuron death, glial activation, blood–brain barrier (BBB) disruption, and oxide stress response in the development of RIBI, the specific mechanisms remain unclear.^5^ Therefore, it is crucial to deepen our understanding of the pathogenesis of RIBI and to explore new targets for disease prevention and treatment.

Adaptive immunity has been involved in the development of many neurological diseases including multiple sclerosis (MS),^9^, ^10^ neuromyelitis optica spectrum disorder (NMOSD),^11^ Parkinson's disease (PD),^12^, ^13^, ^14^ Alzheimer's disease (AD),^15^ ischemic stroke,^16^, ^17^ and hemorrhagic brain injury.^18^ Adaptive immune cells play crucial roles in these diseases through various mechanisms including secreting cytokines and killing targeted cells. While several studies have demonstrated the infiltration of T lymphocytes in the brain as a hallmark of RIBI pathology,^19^ their specific effects on the disease have received limited investigation.

Our recent work has indicated that cytotoxic CD8^+^ T cells (CD8^+^ CTLs) are accumulated by activated microglia through CCL2/8‐CCR2/5 signaling contributing to neuron damage in RIBI, which supports the involvement of infiltrated peripheral lymphocytes in the development of RIBI.^20^ However, as another important T population, CD4 lymphocytes' characteristics in RIBI remain unclear. Of note, apart from CD8^+^ CTLs, a population of CD4^+^ T cells can also acquire cytotoxic ability (CD4^+^ CTLs) and directly kill target cells.^21^ Although the downstream cytotoxic molecules and pathways appear to be common, and some master regulators in their differentiation are shared, there are differences in specific mechanisms and pathological roles between the CD8^+^ CTLs and CD4^+^ CTLs.^21^ In general, the CD8^+^ CTLs mainly target cells expressing major histocompatibility complex class I (MHCI), whereas CD4^+^ CTLs kill MHCII^+^ cells. CD4^+^ CTLs are well characterized as key players in multiple tumors, viral infections, and some autoimmune diseases.^22^, ^23^ Recent studies have also identified CD4^+^ CTLs in neurological disorders such as AD, MS, and PD.^24^, ^25^, ^26^ It has been strongly supported that the CD4^+^ CTLs are a pathogenic CD4^+^ subset driving the progress of MS/EAE, with prognostic values and the potential to serve as a therapeutic target.^27^ Given the presence of CD4^+^ CTLs in other neurological disorders and their potential therapeutic significance, it is reasonable to investigate their role in RIBI. Additionally, CD4^+^ CTLs have been detected in the circulation of supercentenarians with clonal expansion, and a study has found that CD4^+^ CTLs can kill senescent cells, implying their protective effects on aging‐associated diseases.^28^, ^29^ Since there are close relationships between radiation and aging, and radiation is utilized to induce a model of accelerated aging,^30^ it is plausible that CD4^+^ CTLs may be generated in RIBI, and relevant investigation is of clinical significance.

Modern technologies such as single‐cell RNA sequencing (scRNA‐seq) and T‐cell receptor sequencing (TCR‐seq) have enabled detailed and comprehensive studies of the transcriptional profile of immune cells in diseases.^12^, ^31^ In the present study, we mapped the CD4^+^ T cell landscapes of RIBI patients by analyzing the sequencing results of brain samples, and a novel CD4^+^ CTL subcluster was observed with large clonal expansion in RIBI lesions, expressing granzyme B (GZMB) cytotoxic granules in both patients and gamma knife‐irradiated mice. It is the first study illustrating the transcriptional signatures of CD4^+^ T cells and special CD4^+^ CTLs in RIBI, and more importantly, pointing to potential relationships between the CD4^+^ CTLs and the disease.

## 2 MATERIALS AND METHODS

### 2.1 Human tissue sample collection and single‐cell transcriptome data preprocessing

**FIGURE 1:**
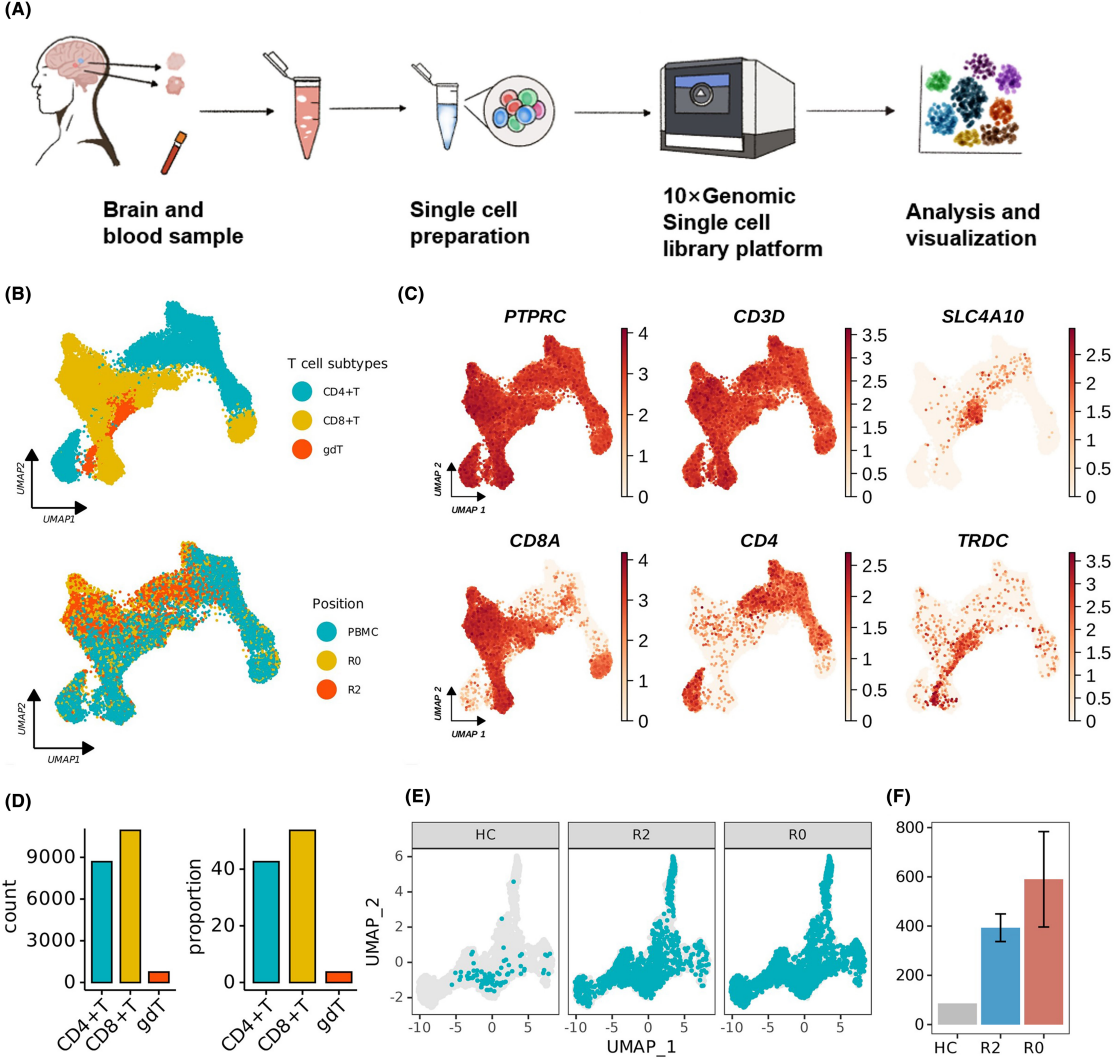
Single‐cell transcriptome profile within CD3^+^ lymphocytes of PBMC and brain tissue from RIBI patients. (A) Flowchart summarizing the single‐cell sequencing steps of isolated cells from RIBI patients. Fresh brain tissues, including proximal lesion (R0) and distal lesion (R2), and peripheral blood from four individuals were analyzed. The samples were sequenced on 10× Genomics single‐cell library platform, and the data were analyzed for subsequent study. (B) Identification of T‐cell subclusters: The uniform manifold approximation and projection (UMAP) plot showing CD3^+^ T cells (*N* = 20,372) from the R0 and R2 of the brain lesion and peripheral blood mononuclear cell (PBMC) of patients with RIBI, colored by cell subtypes (upper) and position (lower). (C) UMAP plot displaying the distributions of canonical marker genes in CD3^+^ T subtypes. (D) Barplot representing cell count (left) and proportion (right) of the three subpopulations within CD3^+^ cells derived from RIBI patients. (E) UMAP plot showing the distribution and number of CD4^+^ T cells in three groups (brain cells from healthy control (HC), R2, and R0). (F) Comparison of CD4^+^ Tcell numbers among HC, R2, and R0 brain samples.

The sample collection and scRNA‐seq procedures were detailed in our previous study,^20^ as shown in the flowchart (Figure 1A). Briefly, brain lesions located in the temporal lobe, including distal lesion (R2) and proximal lesion (R0), as well as peripheral blood samples, were collected from four RIBI patients for a 5′‐end scRNA and scTCR sequencing. Single‐cell suspensions of brain tissue and peripheral blood mononuclear cells (PBMCs) were prepared using the Adult Brain Dissociation Kit (130‐107‐677; Miltenyi Biotec, Germany) and density gradient centrifugation, respectively. Library preparation was constructed with the Chromium Next GEM Single Cell 5′ Library and Gel Bead Kit v1.1 (10× Genomics), along with the Chromium Single Cell V(D)J Enrichment Kit (Human T Cell; 10× Genomics), for 5′ scRNA‐seq and scTCR‐seq. After cell calling, quality control, doublet removal, and batch effect correction were applied, followed by an in‐depth analysis of the sequencing data, including unsupervised clustering and T‐cell identification.

We also re‐analyzed the scRNA‐seq data from healthy controls (HCs) generated by 5′ single‐cell transcriptome and immune repertoire sequencing (GSE181279).^32^ After quality control, data integration was performed using the *RunFastMNN* algorithm provided by the Seurat (version 4.3.0) and SeuratWrappers (version 0.3.1) R packages in R (version 4.2.2).^32^

### 2.2 Cell‐type annotation

To determine the T‐cell composition, we first normalized the raw count data using the *LogNormalize* function. Subsequently, signature genes for every cluster were identified with the *FindAllMarkers* function based on the Wilcoxon algorithm.^33^ T‐cell subtypes were manually annotated by checking canonical marker genes.

### 2.3 Pathway enrichment analysis

To explore the biological function differences in cytotoxic CD4^+^ T cells, genes upregulated in CD4^+^ T_C1 (*log*_*2*_ *(fold change) > 0.25*; *p_val_adj < 0.05*), identified from those differentially expressed between CD4^+^ T subtypes using the *FindAllMarkers* function (*test.use = ‘wilcox’*; *min.pct = 0.3*), were subjected to *enrichGO* provided by the clusterProfiler (version 4.6.0) R package.^34^ The differentially expressed genes (DEGs) from the four phases along pseudotime were input into the *compareCluster* and *simplify* functions using default parameters. Enriched pathways for functional annotation were selected with *pvalueCutoff = 0.01* and *qvalueCutoff = 0.05*.

### 2.4 Single‐cell trajectory analysis of CD4 ^+^ T cell

Monocle2 (version 2.26.0) was used to construct the cell trajectory of CD4^+^ T cells.^35^ Firstly, a CellDataSet object was created, and the raw data were converted to normalized counts using the parameter ‘*expressionFamily = negbinomial. size*’. After calculating size factors, estimating dispersions, and identifying highly variable genes, CD4^+^ T subtypes were performed before ordering the cells, as recommended in the tutorial. After dimension reduction and setting ‘CD4T_C5’ as the starting point of the trajectory, a model of CD4^+^ T‐cell state transition was constructed. The acquired result was visualized using the ‘*plot_cell_trajectory*’ and ‘*plot_pseudotime_heatmap*’ functions.

### 2.5 Transcriptional regulatory network analysis

The construction of the gene regulatory network for CD4^+^ T cells involved four main steps: network inference using GRNBoost2, module generation, regulon prediction using cisTarget, and cellular enrichment analysis using AUCell. All of these methods were implemented using pySCENIC (version 0.12.1).^36^

### 2.6 TCR analysis

TCR sequences were assembled using the '*vdj*' command from Cell Ranger version 6.0.1. The reference genome and annotation utilized were GRCh38‐alts‐ensembl‐5.0.0, obtained from the manufacturer's website. For subsequent analysis, scRepertoire was employed to identify clonotypes based on the combination of CDR3 amino acids.^37^ The STARTRAC R package was conducted to quantify clonal expansion, migration, and state transition potential within different CD4^+^ T subtypes.^38^

### 2.7 Animals

Six‐ to eight‐week‐old wild‐type (WT) male C57BL/6J mice were purchased from the animal facility of Sun Yat‐sen University. All the animal experiments were conducted in accordance with the National Institutes of Health Guide for Care and Use of Laboratory Animals and approved and supervised by the Bioethics Committee of Sun Yat‐sen University. All mice were housed under specific pathogen‐free conditions with a 12‐h light–dark cycle, constant temperature (relative, 22°C), and humidity (relative, 30%), at Sun Yat‐sen University (Guangzhou, China).

### 2.8 Mouse model of gamma knife irradiation

To replicate the RIBI condition observed in patients, mice were subjected to gamma knife irradiation targeted at the unilateral thalamus as previously described.^39^ In brief, mice were anesthetized and restrained on a Leksell stereotactic coordinate Frame G in the prone position, attached to the CT fiducial indicator box. Brain computed tomography (CT) scanning was performed with a CT machine (GE Revolution CT; GE, USA). The scanning voltage was 80 kV. Both the slice thickness and interval were 0.625 mm, the field‐of‐view (FOV) size was 195 mm, the reconstruction matrix was 512 × 512, and the pixel size was 0.38 mm × 0.38 mm × 0.625 mm. The CT image was imported into Leksell GammaPlan (LGP) version 10.2. A 4‐mm collimator was selected, and the irradiation target was defined based on the data (coordinates: anterior–posterior direction (AP) −2 mm, lateral (L) 2 mm, and dorsal–ventral direction (DV) 2 mm relative to bregma and dural surface). Leksell Gamma Knife® Perfexion™ (LGK; Elekta Company, Sweden) was used to perform the gamma knife irradiation. At 8 weeks post‐irradiation, magnetic resonance imaging (MRI) was conducted to validate the modeling effectiveness.

### 2.9 Immunofluorescence staining

Eight weeks after gamma knife irradiation modeling, mice were anesthetized and transcardially perfused with cold PBS, followed by 4% paraformaldehyde (PFA). Brains were extracted and placed into tubes filled with 4% PFA for 1–2 h and cryopreserved with gradient sucrose (20% and 30% sucrose) at 4°C. After being embedded with OCT molds, the mice brains were frozen at −20°C and cut into sections (30 μm thickness) for further immunostaining. A blocking solution with 5% bovine serum albumin (BSA; MRC, USA) and 0.3% Triton X‐100 (Sigma‐Aldrich, Germany) was used to block and permeabilize the frozen sections. For IF labeling, frozen sections were incubated with rat anti‐CD4 (1:200; eBioscience, USA) and GZMB (1:50; Abcam, USA) at 4°C overnight, followed by appropriate Alexa Fluor‐conjugated secondary antibody (1:500; Jackson, USA) at room temperature for 2 h. The brain slices were mounted with VECTASHIELD Antifade Mounting Medium (Vector, USA) with 4′,6‐diamidino‐2‐phenylindole (DAPI) and imaged by fluorescence microscope (Leica DM6B; Germany).

### 2.10 Isolation of cells from the brain and flow cytometry

Brain tissues were placed into 500 ug/ml collagenase type IV (Solarbio, China; dissolved in RPMI 1640 medium containing 10% FBS) and cut into several pieces with a surgical scalpel. After digestion at 37°C for 1 h, cells were pressed through a 70‐μm cell strainer. Myelin debris was removed, and single‐cell suspension was acquired by gradient density centrifugation (30%/70% Percoll solution; GE Healthcare, USA). For surface staining, the brain cells were incubated with CD45‐APC‐Cy7 (Biolegend, USA) and CD4‐FITC (Biolegend, USA) for 30 min. For intracellular staining, cells were fixed and permeabilized with Fixation Buffer (Biolegend, USA) and Intracellular Staining Perm Wash Buffer (Biolegend, USA) according to the manufacturer's guidelines, followed by GZMB‐APC/Per‐Cy7 (Biolegend, USA) staining for 40 min. The eBioscience™ Foxp3/Transcription Factor Staining Buffer Set (00‐5523‐00; Thermo Scientific) and Foxp3‐PE antibody (Biolegend, USA) were utilized for intranuclear staining in Treg cells. Flow cytometry analysis was performed by CytoFLEX cytometer (Beckman, CA). The data were analyzed with CytExpert and FlowJo software.

### 2.11 Western blotting

Ipsilateral thalamus from mice was acquired at 8 weeks after irradiation and stored at −80°C for further assessment. Brain tissues were lysed with RIPA lysis buffer (P0013B; Beyotime) on ice for 20 min. After centrifugating at 15,000 rpm for 15 min, the supernatants were transferred to a new EP tube. The BCA Protein Assay Kit (23227; Thermo Scientific) was used to assess the total protein concentration in every sample. The protein samples were loaded on 10% gradient sodium dodecyl sulfate–polyacrylamide gels, transferred onto polyvinylidene difluoride membrane, and blocked with 5% non‐fat milk. The membranes were incubated with anti‐β‐tubulin (10068‐1‐AP‐100UL, 1:5000; Proteintech) and anti‐caspase‐3 (9662S, 1:1000; CST) overnight, followed by goat anti‐rabbit HRP at room temperature for 1 h. Detection was performed using the Odyssey Infrared Imaging System (LI‐COR Biosciences, Lincoln, NE, USA), and the bands were quantified by ImageJ software.

### 2.12 Statistical analysis

Bioinformatic analysis was carried out with the R software (version 4.2.3), as previously described. Experimental data analysis was performed using the GraphPad Prism 8.0 software. The Shapiro–Wilk test was used to evaluate the normality of the datasets. If the data passed the normality test, unpaired two‐tailed Student's *t*‐tests were used to compare differences between the two groups. Alternatively, when the data did not follow a normal distribution, a chi‐square test was employed. Continuous variables were expressed as the median (range) or mean ± SEM. Statistical significance was considered at *p* < 0.05.

## 3 RESULTS

### 3.1 Single‐cell RNA sequencing (scRNA‐seq) revealed altered cellular immune response in RIBI patients

As described in our previous studies and shown in the flowchart, single cells from PBMC and paired brain tissues (R0 and R2) of four RIBI patients diagnosed were collected and sequenced (Figure 1A). To explore the signature of adaptive immune cells in RIBI, we extracted T‐cell cluster with canonical marker gene expression (PTPRC, CD3D). Uniform manifold approximation and projection (UMAP) dimension reduction identified three populations including CD4^+^ T, CD8^+^ T, and γδT cells within the T‐cell gating (Figure 1B,C). In a total of 20,372 CD3^+^ T cells, the count and proportion of CD4^+^ and CD8^+^ T cells occupied the two major populations, while γδT cells occupied the minor one (Figure 1D). Our previous study has focused on the roles of CD8^+^ T cells in RIBI.^20^ To further confirm the CD4^+^ T‐cell‐mediated immune response in RIBI patients, we re‐analyzed previously published data from HC samples. As shown in Figure 1E,F, compared with the HCs, the CD4^+^ T cells were significantly accumulated in the brain tissue (both R0 and R2) of RIBI patients. The above findings support the infiltration of CD4 clusters in the brain lesions.

### 3.2 Validation of CD4 ^+^ T‐cell CNS infiltration in RIBI patients and gamma knife‐irradiated mice

**FIGURE 2:**
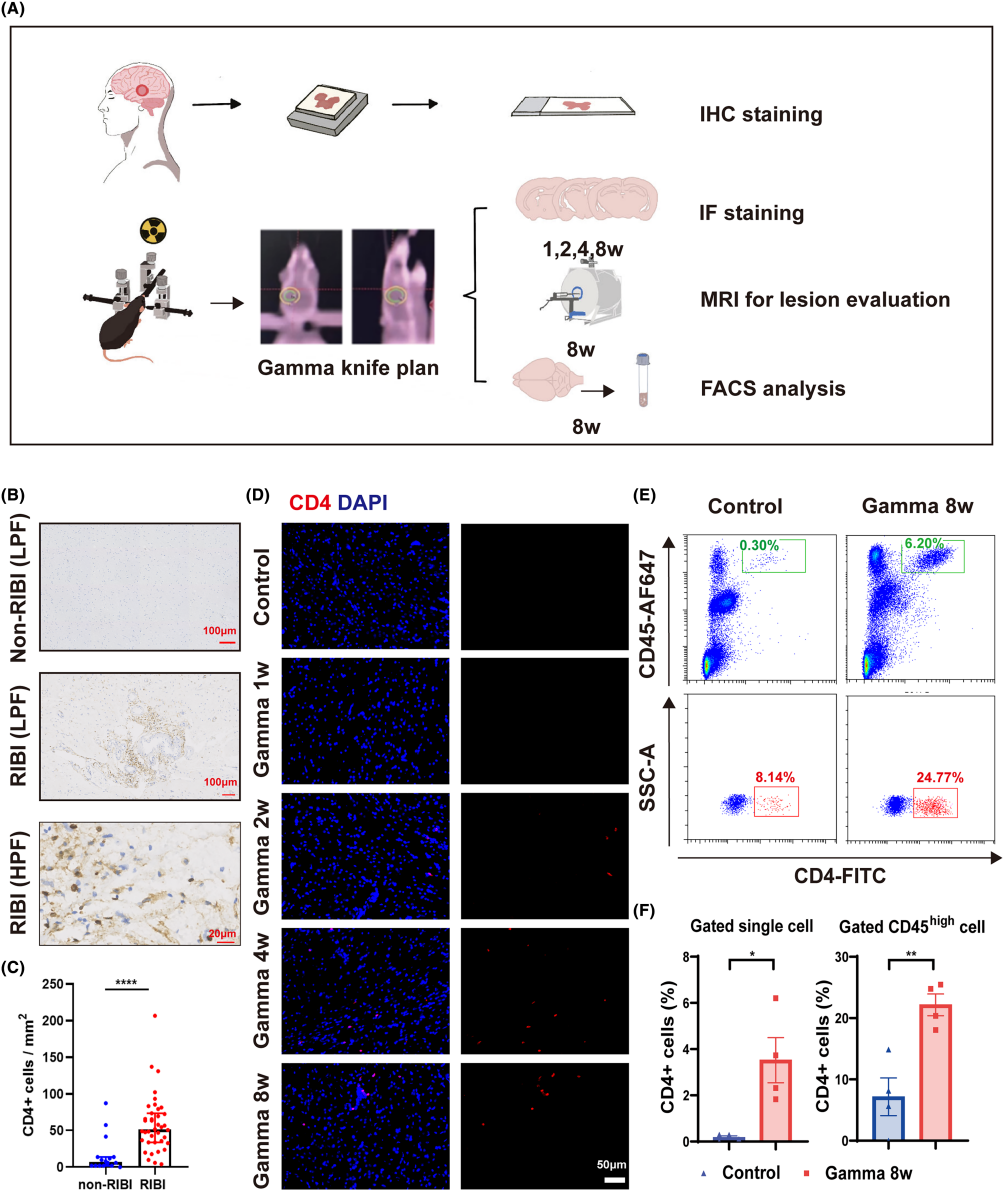
Validation of CD4^+^ T‐cell CNS infiltration in both RIBI patients and gamma knife irradiation mouse model. (A) The experimental flowchart illustrated the validation methods with clinical samples and animal models. In animal experiments, mice were irradiated with gamma knife on the left thalamus. MRI was performed post‐8 weeks to ensure the validity of the simulation model. Brain sections were stained for CD4 assessment. FACS analysis for brain single cells was carried out at 8 weeks post‐irradiation. (B) Representative IHC staining pictures evaluating the CD4 protein expression in non‐RIBI and RIBI patient brains (scale bar = 50 μm). (C) Quantification of CD4‐immunoreactive cells in brain tissue from non‐RIBI and RIBI patients. (D) Immunofluorescent staining of CD4 (red) and DAPI (blue) in mouse brain (lesion site) from the control group and gamma knife‐irradiated group at 1 week, 2 weeks, 4 weeks, and 8 weeks after irradiation (scale bar = 50 μm). (E) Scatter plots of FACS analysis in brain single‐cell suspension. The CD45^hi^ CD4^+^ cells within the single cell (upper) and CD4^+^ cells within the CD45^hi^ gating (lower) were highlighted with green and red borders, respectively. (F) The histograms show the comparison of CD4^+^ cell proportion in single cell (left) and in CD45^hi^ gating (right) between the control and gamma knife‐irradiated mice with flow cytometry analysis. Data were presented as mean ± SEM, and every point represented results from an individual. All the data passed the Shapiro–Wilk normality test, and unpaired two‐tailed Student's *t*‐tests were conducted for two group comparisons (*p < 0.05, **p < 0.01, ****p < 0.001).

Since the results of scRNA‐seq determined the CD4^+^ T‐cell‐mediated immune response in CNS of RIBI patients, we thus conducted preliminary clarification (shown in the flow diagram, Figure 2A). In the patient level, IHC staining showed a significant increase of CD4^+^ T cells in the lesion core of RIBI patients compared with the brain of HCs (Figure 2B,C).

Gamma knife irradiation on the unilateral thalamus has been reported as an effective animal model for simulating RIBI damage in humans.^39^ At 8 weeks after gamma knife irradiation, the mice showed high signal intensity in the target region on contrast‐enhanced T1WI sequencing, compared with the control group (Figure S1A), indicating effective modeling. The time‐course evaluation for CD4^+^ T cell was conducted in brain sections collected at 1 week, 2 weeks, 4 weeks and 8 weeks post‐irradiation group and control mice. While almost no CD4^+^ T cells were displayed in the brain from 1 week post‐irradiated mice like the control group, the CD4^+^ T lymphocytes started to accumulate in the lesion site from 2 weeks after irradiation and the cell number increased over time (Figure 2D,E). Simultaneously, MHCII^+^ cells were dramatically increased and gathered around the lesion site of mouse brains (Figure S1B). We also utilized the flow cytometry for validation, and the results showed that compared with the control group, the mice at 8 weeks post‐irradiation were presented with a significant increase of CD45^hi^ cells (generally regarded as infiltrated lymphocytes) in the brain. Within the lymphocyte gating, the CD4^+^ T cells with FITC labeling antibody were detected. Comparison between the two groups confirmed a statistically significant higher proportion of CD4^+^ cells in the irradiation group than in the control group (Figure 2F). Moreover, the proportion of CD4^+^ T cells evaluated within the single‐cell gating also supported the accumulated CD4^+^ T cells in the RIBI. Taken together, we validated the CNS accumulation of CD4^+^ T cell total population in both RIBI patients and animal models.

### 3.3 Subcluster analysis identified a specific CD4 ^+^ subtype that expresses cytotoxic molecules (CD4 ^+^ CTL) in RIBI patients

**FIGURE 3:**
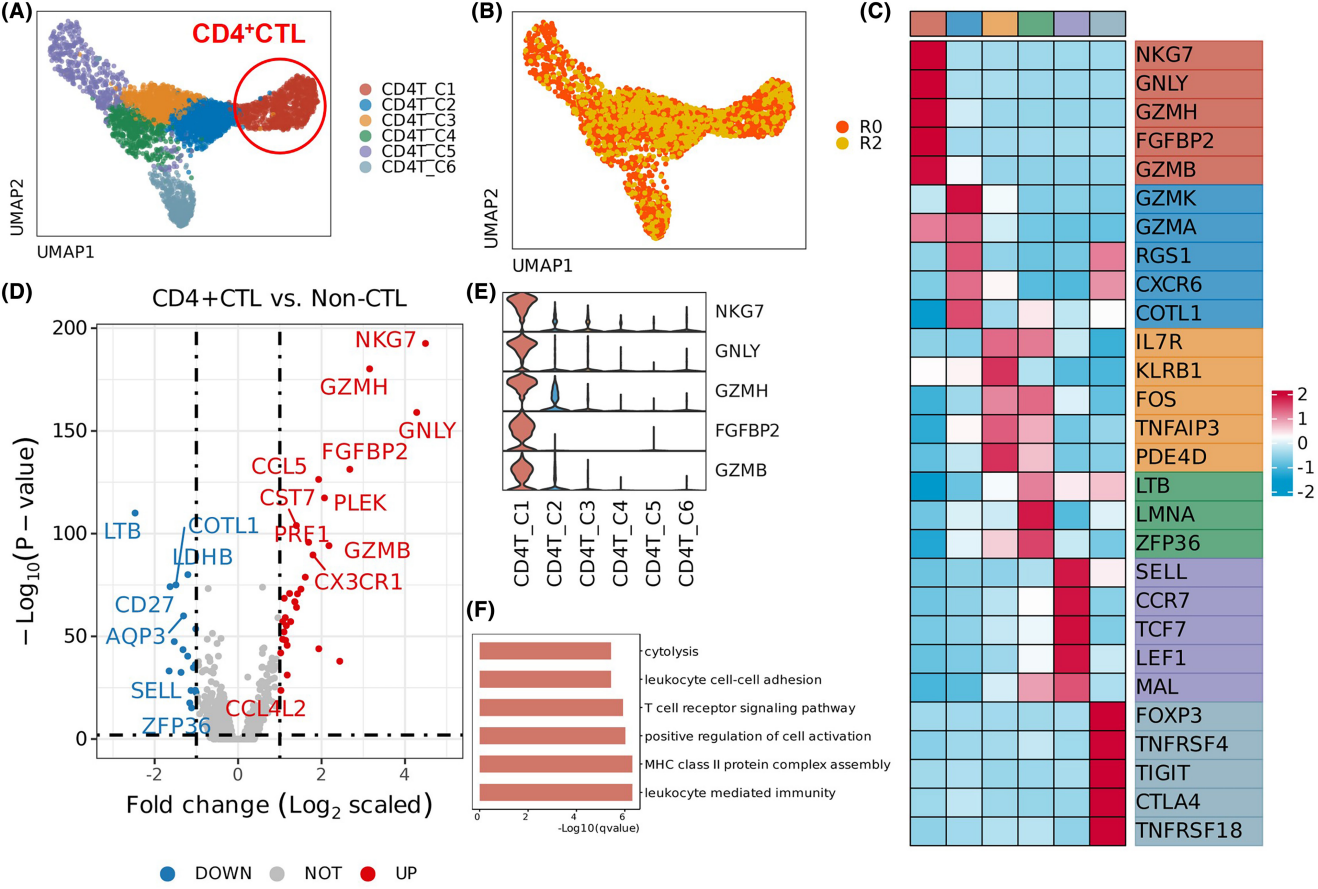
Single‐cell transcriptional analysis within CD4^+^ T cells in RIBI patients identified six clusters including a special CD4^+^ CTL subset. (A) UMAP visualization of subclusters within CD4^+^ T cells (*N* = 3934) from brain tissue (R0 and R2) of RIBI patients. (B) Position distribution (R0 colored with red and R2 colored with yellow) of CD4^+^ T cells in RIBI patient brain with UMAP plot. (C) Heatmap displaying *z* scores of the average expression of select marker genes for the annotated cluster in (A). The upper color panel represents different subtypes. (D) Volcano plot showing differentially expressed genes (DEGs) with adjusted *p*‐value < 0.01 and log_2_ (fold change) > 1, comparing CD4^+^ CTLs (C1, red dots) to non‐cytotoxic CD4^+^ T cells (blue dots). (E) Violin plot displaying the expression of selected cytotoxic effector genes in CD4^+^ T subclusters. (F) Barplot illustrating the pathways significantly enriched in CD4^+^ CTLs, based on upregulated genes.

It is well known that CD4^+^ T cells are categorized into several subsets such as Th1, Th2, Th17, and Treg, with distinct transcriptional and cytokine profiles exerting specific effects in various diseases. Increasing evidence has also revealed a novel subset CD4^+^ CTL under certain conditions, and we speculated that it may also be induced in RIBI. Therefore, after validation of CD4^+^ T‐cell infiltration in the RIBI brain, we explored their transcriptional signatures and conducted unsupervised sub‐clustering analysis. We identified six transcriptionally distinct clusters within the CD4^+^ T population as shown in Figure 3A,B. The heatmap showed the marked genes differentially expressed in the six clusters (Figure 3C). Strikingly, molecules associated with cytotoxic functions including *NKG7*, *GNLY*, *GZMH*, *FGFBP2*, and *GZMB* were highly expressed in the *CD4_cluster1* (defined as CD4^+^ CTLs in the present study), and *GZMK*, *GZMA*, *RGS1*, *CXCR6*, and *COTL1* were markers for *CD4_cluster2*. The *CD4_cluster3* was identified with *IL‐7R*, *KLRB1*, *FOS*, *TNFAIP3*, and *PDE4D*, and the *CD4_cluster4* was identified with *LTB*, *LMNA*, and *ZFP36*. The *CD4_cluster5* expressed high levels of *SELL*, *CR7*, *TCF7*, *LEF1*, and *MAL*, showing the Naïve CD4^+^ T characteristics, while the *CD4_cluster6* highly expressed conventional Treg hallmark genes and immunoregulatory genes, including *Foxp3*, *TNFRSF4*, *TIGIT*, *CTLA‐4*, and *TNFRSF18*.

Since the CD4^+^ T cells were traditionally considered as a helper in the adaptive immune response, the discovery of CD4^+^ CTLs showing direct kill potential in several diseases has recently attracted increasing attention.^21^ However, the characteristics of CD4^+^ CTLs exist in heterogeneity among diseases. We thus focused on the *CD4_cluster1* and explored its expression profiles in RIBI (Figure 3D,E). The differentially expressed gene (DEG) analysis revealed that compared with the non‐CTL, the cytotoxicity‐related genes including *NKG7*, *GZMH*, *GNLY*, *FGFBP2*, *GZMB*, and *PRF1* were significantly abundant in the CD4^+^ CTL cluster, accompanied by upregulated chemokine and chemokine receptors including *CCL5*, *CX3CR1*, and *CCL4L2*. We also found significant downregulation of *COTL1*, *LDHB*, *CD27*, *LTB*, *AQP3*, *SELL*, and *ZEP36* molecules in CD4^+^ CTLs, distinct from other CD4^+^ subsets (non‐CTL). Based on these significant molecules of CD4^+^ CTLs, the pathway analysis was conducted and showed enrichment in cytolysis, leukocyte cell–cell adhesion, T‐cell receptor signaling pathway, positive regulation of cell activation, MHC class II protein complex assembly, and leukocyte‐mediated immunity pathways (Figure 3F).

### 3.4 The CD4 ^+^ CTLs (CD_cluster1) in RIBI exhibited a terminal differentiation state

**FIGURE 4:**
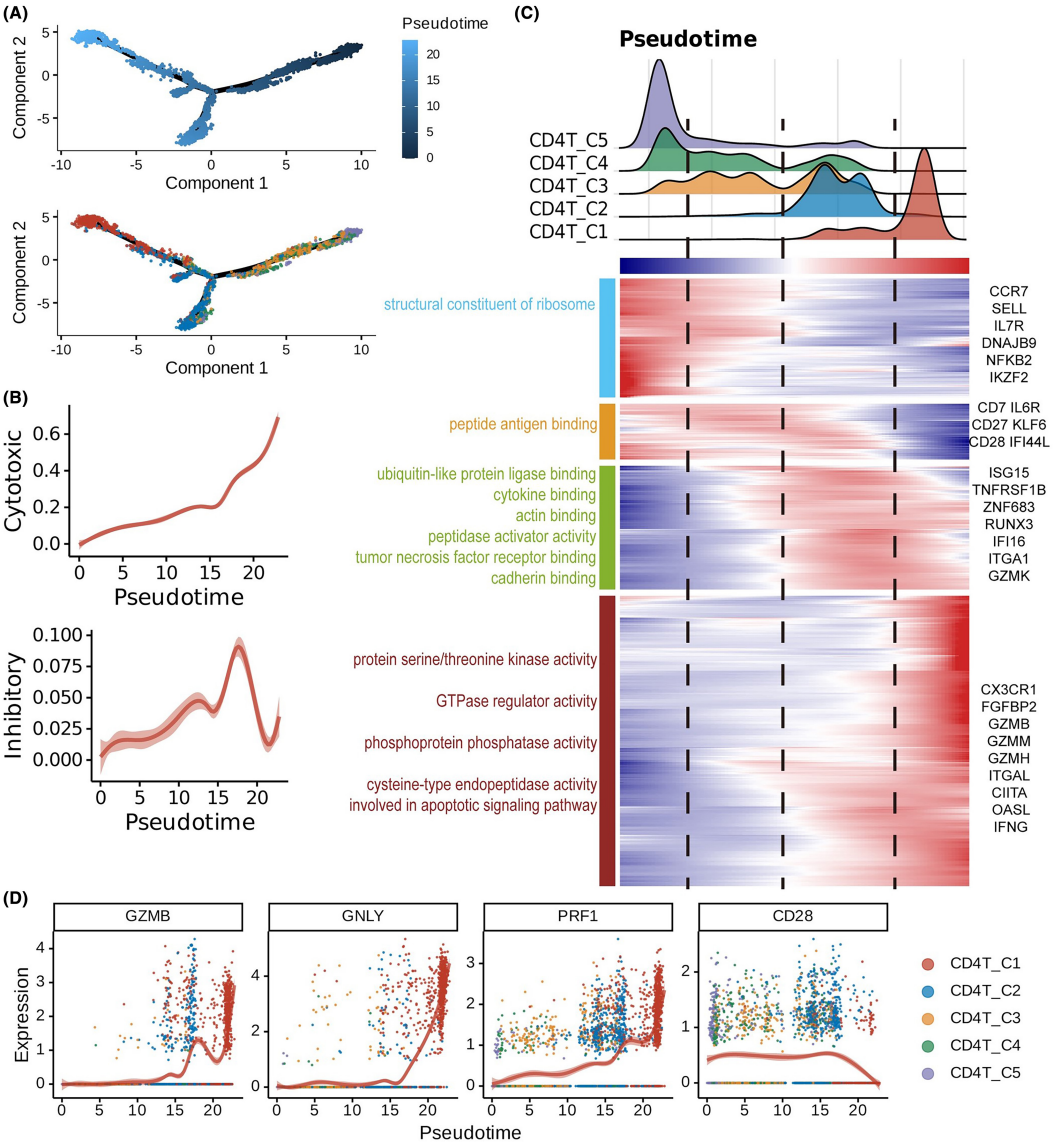
Pseudotime analysis identified the terminal differentiation signatures of CD4^+^ CTLs in RIBI patients. (A) The pseudotime trajectory illustrates the dynamic states of CD4^+^ T cells estimated using Monocle2. Each dot represents an individual cell and is color‐coded based on pseudotime (upper) and cell types (lower). (B) Two‐dimensional plots reveal the changes in expression scores for cytotoxicity (upper) and inhibitory receptor (right) signatures during the CD4^+^ T‐cell transitions. Error bands show local polynomial regression and the 95% confidence interval, respectively. (C) Heatmap showing the dynamic expression of genes (lower panel) and the distribution of CD4 subtypes along the pseudotime divided into 4 phases (vertical dashed line). The upper panel labels the subtypes by colors. The pathways enriched in different phases are listed on the left side, and marker genes are annotated on the right side. (D) The dynamic expression of selected cytotoxic genes (*GZMB*, *GNLY*, and *PRF1*) and differentiation‐associated gene *CD28*. Each dot represents a single cell and is colored by CD4 subtypes.

With scRNA‐seq analysis, we detected CD4^+^ CTLs within the CD4^+^ T‐cell population in RIBI, implying their involvement in the development of RIBI. To better understand the differentiation signatures and potential effects of this special CD4^+^ T subcluster, we utilized pseudotime ordering analysis to simulate the differentiation routes of CD4^+^ T cells from brain tissues of RIBI patients. The main trajectory was identified as originating from Naïve CD4^+^ T cells (*CD4_cluster5*), progressing toward *CD4_cluster2*, and terminating at CD4^+^ CTL (*CD4_cluster1*), with a branch out (Figure 4A). We also observed a gradual increase of cytotoxic and inhibitory scores accompanied by the differentiation of CD4^+^ T cells (Figure 4B). We mapped the marked genes and enriched pathways in different states of CD4^+^ T cells. The end state composed the majority of CD4^+^ CTLs, which was marked with *CX3CR1*, *FGFBP2*, *GZMB*, *GZMM*, *GZMH*, *ITGAL*, *CIITA*, *OASL*, and *IFNG* genes. Functional enrichment analysis showed that the CD4^+^ CTLs at terminal differentiation state were abundant in protein serine/threonine kinase activity, GTPase regulator activity, phosphoprotein phosphatase activity, and cysteine‐type endopeptidase activity that is involved in the apoptotic signaling pathway (Figure 4C). Consistently, the expression of *GZMB*, *GNLY*, and *PRF1*, marked molecules of CD4^+^ CTLs, was increased along the latter half of pseudotime, while the expression of co‐stimulatory molecule *CD28* was decreased and almost negative in the terminal differentiation state (Figure 4D). Taken together, the CD4^+^ CTL subcluster in RIBI brain was identified at a terminal differentiation state, capable of the highest cytotoxicity.

### 3.5 The CD4 ^+^ CTLs were detected in the brains of gamma knife irradiation mice

**FIGURE 5:**
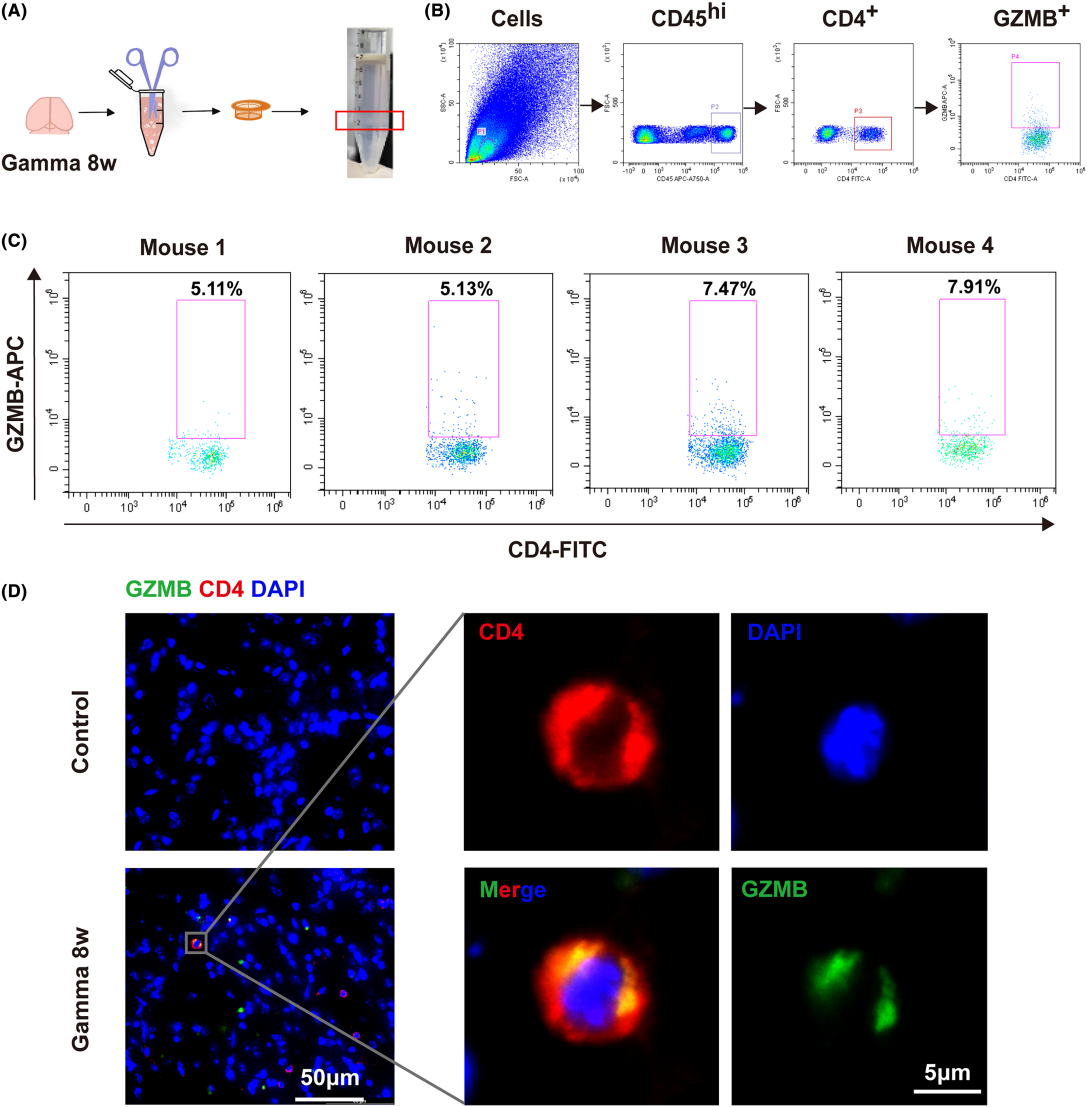
GZMB‐producing CD4^+^ CTLs were presented in RIBI. (A) At 8 weeks post‐gamma knife irradiation, single‐cell suspensions of mouse brain tissue were obtained and subjected to flow cytometry analysis. Before GZMB intracellular staining, the cells were fixed and permeabilized. (B) Representative flow cytometry gating strategy. From left to right, the gated cells are as follows: the target population (P1) in all events, the CD45^hi^ cells within the P1 gating as an infiltrating lymphocyte group (P2), the CD4^+^ cells under the CD45^hi^ cells (P3), and GZMB‐producing cells within the CD4^+^ population (P4). (C) Scatter plots displaying the CD4^+^GZMB^+^ cells within the CD4^+^ gating in the brain samples from 4 gamma knife‐irradiated mice. (D) Co‐location of GZMB (green) and CD4 (red) cells in mouse brain after irradiation with immunofluorescence staining.

The characteristics and possible development routes of CD4^+^ CTLs were displayed at the transcriptional level. We next sought to determine whether the CD4^+^ CTLs were presented in RIBI. We applied flow cytometry to evaluate the GZMB expression in CD4^+^ T cells (shown in flowchart Figure 5A), and the result confirmed the presence of CD4^+^ CTLs in mouse brains at 8 weeks after gamma knife irradiation (Figure 5B,C). Additionally, the GZMB was also expressed in CD3^+^CD4^−^ T cells, which are primarily CD8^+^ cells (Figure S2A,B), while minimal GZMB signals were observed in CD4^+^Foxp3^+^ Tregs in brain tissue from RIBI mice, suggesting that CD4^+^ CTL represents a novel subtype distinct from Tregs (Figure S2C). Moreover, the proportion of CD4^+^ CTLs was also elevated in both peripheral blood and spleen of RIBI mice (Figure S2D,E). Subsequent immunofluorescence assays also showed co‐localization of GZMB and CD4^+^ T cells in brain slices of irradiated mice (Figure 5D). Since CD4^+^ CTLs can kill MHCII^+^ target cells mediating programmed cell death process known as apoptosis, we preliminarily assessed the level of apoptosis‐associated proteins. Exposure to gamma knife irradiation induced increased expression of caspase‐3 and cleaved caspase‐3 in mouse unilateral thalamus (Figure S3A,B). Our results validated the existence of CD4^+^ CTLs in RIBI, which may affect the progress of RIBI through inducing cell apoptosis of MHCII^+^ target cells.

### 3.6 The CD4 ^+^ CTLs exhibited large clonal expansion in RIBI with master transcriptional factors (TFs)

**FIGURE 6:**
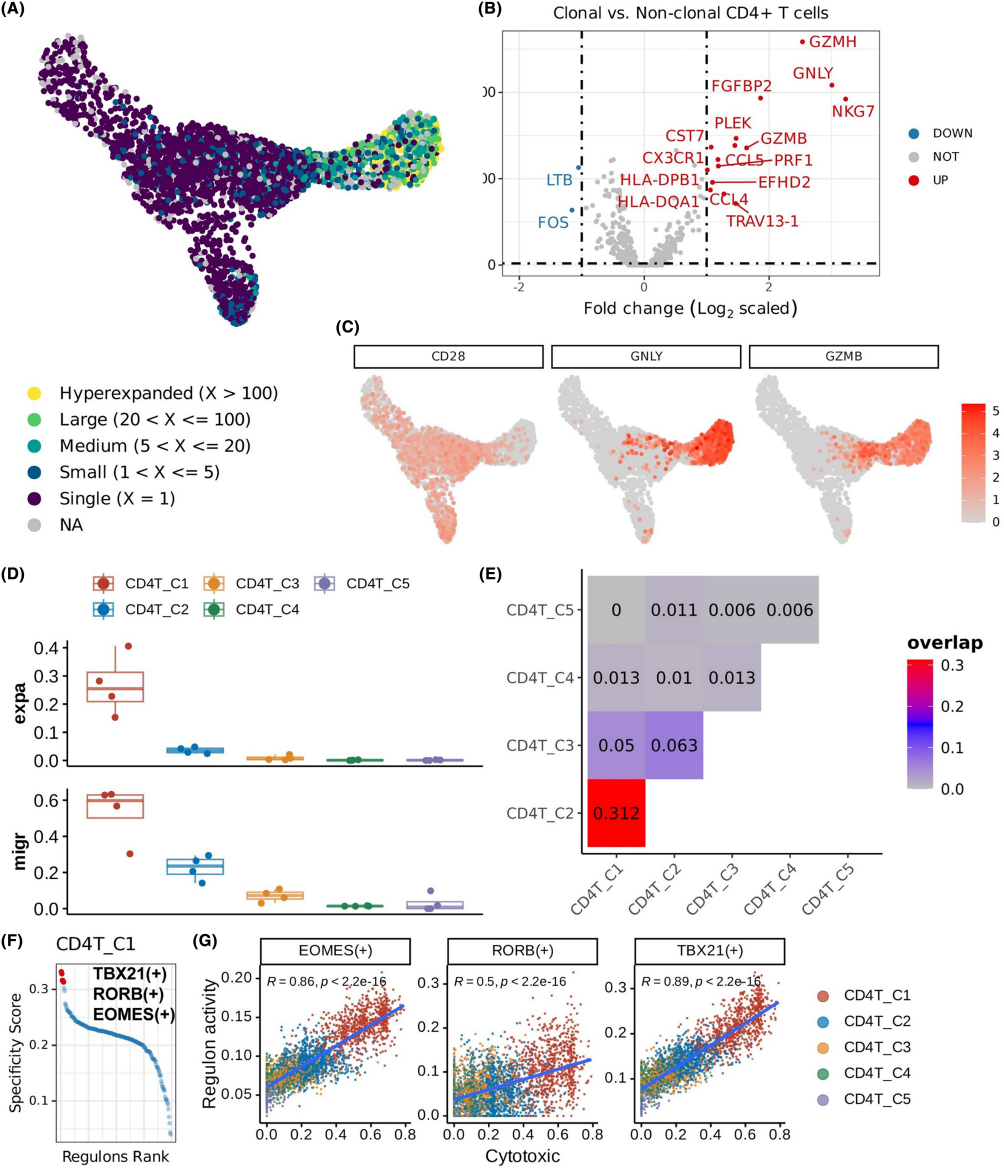
TCR sequencing analysis showed a large clonal expansion of CD4^+^ CTLs in RIBI patients and their potential key TFs. (A) UMAP embedding on CD4^+^ T cells illustrating the TCR clonal size. Each dot in the graph represents a single cell, and the color of each dot indicates the frequency of the corresponding clonotype. (B) Volcano plot showing DEGs with adjusted *p*‐value < 0.01 and log_2_ (fold change) > 1, comparing clonal (red dots) to non‐clonal CD4^+^ T cells (blue dots, excluding the CD4_cluster6). (C) The expression of *CD28, GNLY,* and *GZMB* in CD4^+^ T cells visualized by UMAP plot. (D) Barplot showing the clonal expansion (expa) and tissue migration (migr) of CD4^+^ T subtypes assessed by Single T‐cell Analysis by Rna‐seq and Tcr TRACking (STARTRAC) methodology. (E) The heatmap visualizing the measurement of clonal similarity between different CD4 clusters using the overlap coefficient. (F) Scatter plot depicting specialized transcription factors (TFs) within CD4^+^ CTLs. Each point represents a regulon. The top 3 regulons are emphasized and visually highlighted in red. (G) Correlation analysis of TF regulon activity and cytotoxic scores in the CD4^+^ T cells of RIBI patients. Subtypes are labeled with different colors. Each point represents a cell.

Based on previous results, we hypothesized that the CD4^+^ CTLs may be clonally expanded in response to potential antigens after irradiation exposure and act as pathogenic CD4^+^ subset in RIBI. We thus performed scTCR‐seq to gain insight into the clonal expansion of CD4^+^ T cells in the RIBI. Cells isolated from the patient lesion (R0 and R2) that share the same CDR3 amino acid sequences in both TCR‐α and TCR‐β chains were defined as clonal. According to UMAP projection, the CD4^+^ CTL cluster was confirmed to exhibit prominent clonal expansion at the lesion site of RIBI patients, marked with low expression of *CD28* molecules but high expression of cytolytic molecules such as *GZMB* and *GNLY* (Figure 6A–C). In addition, a comparison of transcription profiles between the clonally expanded CD4^+^ and non‐clonal CD4^+^ T cells was conducted. And the results showed that molecules related to cytotoxic function and immune response including *GZMH*, *GNLY*, *NKG7*, *FGFBP2*, *PLEK*, *GZMB*, *PRF1*, *CST7*, *CCL4*, *EFHD2*, *HLA‐DPB1*, *HLA‐DQA1*, *TRAV13‐1*, and *CCL5* were enriched in the clonally expanded CD4^+^ T cells, while *LTB* and *FOS* genes were downregulated compared with unexpanded CD4^+^ T cells (Figure 6B). In addition, the STARTRAC migration and expansion indexes revealed that CD4^+^ CTLs were capable of the highest mobility and proliferative frequency among all CD4 clusters (Figure 6D). Collectively, these observations indicated the clonal expansion of CD4^+^ CTLs in RIBI, which implies that this cluster may experience antigen activation and exert direct cytotoxic effects in this disease. Furthermore, as presented in the heatmap (Figure 6E), the CD4^+^ CTL (*CD4_cluster1*) cluster had overlapped TCR sequences with other CD4^+^ T cells except the *CD4_cluster5*. Notably, *CD4_cluster2* showed the most similarity in clones with CD4^+^ CTLs, suggesting a tight linkage between the two subsets in the development of RIBI.

Transcriptional factors (TFs) are crucial for the differentiation from Naïve CD4^+^ T cells toward effector subsets. With correlation analysis between known regulons (transcriptional factors and their targets) and cytotoxicity in CD4^+^ T cells, we found that *TBX21*, *RORB*, and *EOMES* TFs showed positive correlations with cytotoxicity of CD4^+^ T cells and acted as master regulators to distinguish CD4^+^ CTLs from other CD4 subsets (Figure 6F,G). To further compare the CD4^+^ CTLs in RIBI with those in other neurological diseases, we summarized related clinical and animal investigations in Tables 1 and 2. We re‐analyzed scRNA‐seq data of CD4^+^ T cells in PD, AD, and MS to explore the TF profiles of CD4^+^ CTLs among diseases. As shown in Figure S3C, the *ETV1*, *CEBPD*, and *TBX21* marked CD4^+^ CTLs in MS, *NFIL3*, *TFAP2A*, and *TCF7L2* marked PD‐related CD4^+^ CTLs, and *EOMES*, *SPR*, and *HOXA10* were key TFs driving the development of CD4^+^ CTLs in AD. It suggests that the TF signatures of CD4^+^ CTLs in our study may be specific to RIBI.

**TABLE 1:** Clinical investigations of CD4^+^ CTL in CNS diseases.

Disease	Samples and methods	Markers	Other DEGs	Effects
PD^12^	PBMC + CSF (scRNA‐seq)	GZMA, GZMB, GZMH, NKG7 (mainly in PBMC)	VLA‐4, LFA‐1, Mac‐1; CX3CR1, CCL4, CCL5, CD99, SELPLG	Peripheral CD4^+^ CTLs may be differentiated from Th1 cell and be a source of central infiltrating CD4^+^ CTLs involved in the disease pathogenesis
PD^50^	PBMC (scRNA‐seq)	NKG7, GNLY, GZMH	IFNG, CCL5, ITGB2, HLA‐DRB1, CD74 TF: TBX21, IRF1, ASCL2, NFATC2	Peripheral CD4^+^ CTLs might be recruited by *SPP1* and secrete cytokines IFN‐γ to activate ECs, showing potential to destroy the BBB and promote the course of PD
NMOSD^51^	Blood (FACS)	GZMB		Case report
MS^52^	PBMC (multiparametric flow cytometry)	GZMA, EOMES		The increase of CD4^+^ CTLs was more obvious in older patients. Trajectories of CD4^+^ CTLs in the circulation of MS seemed different from those in healthy control
MS^24^	PBMC	CD28 negative		The number of CD4^+^ CD28 null cells was associated with disease severity, which may help predict the disease progress
SPMS^27^	PBMC + brain (flow cytometry)	GZMB, EOMES		The CD4^+^ CTL population was increased in SPMS in the progressive disease phase, with potential as a biomarker and therapeutic target
SPMS^53^	PBMC + CSF	EOMES, CD107a, GZMB, PRF1, IFN‐γ		Eomes^+^ T cells may be a biomarker in the development of RRMS into SPMS
MS^45^	Brain (IHC)	NKG2C		The MBP‐specific NKG2C^+^ CD4 T cell displays a cytotoxic profile (GZMB, PRF1, NKG2D), and it infiltrates the inflamed CNS of MS patients

Abbreviations: CD4^+^ CTL, cytotoxic CD4 T cell; CSF, cerebrospinal fluid; IHC, immunohistochemistry; MS, multiple sclerosis; NMOSD, neuromyelitis optica spectrum disorder; PBMC, peripheral blood mononuclear cell; PD, Parkinson's disease; scRNA‐seq, single‐cell RNA sequencing; SPMS, secondary progressive multiple sclerosis.

**TABLE 2:** Involvement of CD4^+^ CTLs in animal models with neurological diseases.

Model	Samples and methods	Markers	Effects
5 × FAD (AD), mSOD1 (ALS), EAE (MS)^26^	Spinal cord + brain (flow cytometry + ELISpot)	GZMB, EOMES	Eomes^+^ Th cells infiltrated in the CNS of three mouse models with the capacity to secrete GZMB
MPTP mice and DSP‐4 rats (PD)^25^	Splenocyte (flow cytometry + IF)	GZMB, PRF1	CD4^+^ CTLs may induce apoptotic pathways that kill neurons; calpain inhibition reduced the CD4^+^ CTLs in model mice
EAE (MS)^46^	CNS (flow cytometry)	EOMES, CD107a	Protein ORF1 stimulated the degranulation of Eomes^+^ Th cells after ingestion and presentation in the MHC‐II context by microglia. Inhibition of microglia activation decreased the Eomes^+^ Th cells
EAE (MS)^54^	CNS + LN (IF)	CXCR6, GZMC, GZMA, IFN‐γ, GM‐CSF, IL‐17A, IL‐23R. IL‐7a, IL‐1R1	Sb1/1‐ mice are resistant to EAE with decreased CD4^+^ cells that produced molecules listed at the left. Anti‐CXCR6 could deplete this population and ameliorate the EAE
EAE (MS)^55^	Brain (flow cytometry) FACS‐sorted splenic CD226^+^CD4^+^ T cells	EOMES, CD107a	The APCs produced PRL to induce Eomes Th cells. Depletion of B cells ameliorated the EAE accompanied by a decrease in Eomes^+^ Th cells

Abbreviations: AD, Alzheimer's disease; ALS, amyotrophic lateral sclerosis; APCs, antigen‐presenting cells; CD4^+^ CTL, cytotoxic CD4 T cell; CNS, central nervous system; EAE, experimental autoimmune encephalomyelitis; IF, immunofluorescence; LN, lymph node; MS, multiple sclerosis; PD, Parkinson's disease.

## 4 DISCUSSION

Accumulating evidence has supported tight interactions between the CNS and the immune system in neurological diseases, leading to new diagnostic and therapeutic strategies for patients. Previous studies have suggested altered immune responses in RIBI patients and emphasized the nonredundant roles of adaptive immune cells (focusing on CD8^+^ lymphocytes) in the development of RIBI. Herein, combined with bioinformatic analysis and experiments, we clarified the infiltration of CD4^+^ cells in the CNS of RIBI and mapped its transcriptional subpopulations. Of note, a clonally expanded CD4^+^ CTL subset was revealed in both the periphery and lesion of RIBI, characterized by a terminal differentiation state and high expression of cytolytic‐related genes. Moreover, several master regulators were pointed out for the identification of RIBI‐associated CD4^+^ CTLs.

In healthy CNS, the CD4^+^ T cells, although at a low number, are crucial for the maturity and function of microglia, affecting memory, behavior, and other functions of the brain.^40^ Under pathological conditions, peripheral CD4^+^ T cells can be recruited by activated glia cells, infiltrated inflammation cells, injured neurons, or endothelial cells and produce cytokines to mediate CNS inflammation after re‐activation by MHCII^+^ antigen‐presenting cells (APCs).^41^ Since irradiation promotes the release of autoantigens or new antigens, it is rational that brain irradiation induces unknown antigens that can be recognized by APCs, which further triggers the neuroimmunology processes as previously mentioned.^32^ In RIBI patients and gamma knife irradiation mice, the CD4^+^ T cells accumulated time‐dependently after brain irradiation, accompanied by gathered MHCII^+^ cells. It is in line with previous reports in RIBI, although the time points the cells begin to infiltrate seemed different owing to the differences in modeling methods.^19^, ^42^ It suggests the CD4^+^ T‐cell CNS infiltration is a vital pathological component of RIBI, supporting the participation of adaptive immunity in this disease and providing clues for exploring its pathogenesis. Previous studies have also found associations between the level of CD4^+^ T cells and disease severity in MS, PD, stroke, AD, and other neurological diseases,^14^, ^15^, ^16^ so the count of CD4^+^ T cells altered in the periphery or CNS may be an effective index for monitoring the disease process for RIBI patients. Of note, through which path the CD4^+^ T cells migrate to the brain parenchyma and where they are re‐activated remain debated. It is worthwhile to explore these mechanisms with RIBI, and the gamma knife irradiation model used herein is hopeful to be a new animal model for tracking the route of immune cells accumulated into the brain.

In‐depth analysis with scRNA‐seq and TCR‐seq identified a special CD4^+^ subset with cytotoxic capacity clonally expanded in RIBI patients. There existed two CD4^+^ subsets expressing cytotoxicity‐related genes, but the pseudotime analysis revealed *CD4_cluster1* as the terminal differentiation cluster with the highest cytotoxic ability (defined as CD4^+^ CTLs in the present study). According to previous studies, repeated stimulation with the same antigens may induce the differentiation from Naïve CD4^+^ T cells or other CD4^+^ subsets toward CD4^+^ CTLs.^23^ It implies this cluster as possible RIBI‐associated CD4^+^ CTLs responding to undetermined antigens and contributing to chronic inflammation after radiation exposure. The presence of CD4^+^ CTL has been mostly reported in tumors, viral infections, aging, and autoimmune diseases,^22^, ^43^ while the knowledge of CD4^+^ CTL in CNS diseases is relatively lacking (as summarized in Tables 1 and 2). Our observations help further understand the characteristics of CD4^+^ CTLs in neurological disorders. The CD4^+^ CTLs in RIBI were marked with negative or low expression of *CD28* surface protein but high expression in cytolytic molecules such as *GZMB*, *PRF1*, and *GNLY*, in accordance with previous reports in other disorders.^44^ The mechanism by which CD4^+^ CTLs kill target cells involves the production of cytotoxic granules containing PRF1 and apoptosis‐inducing serine proteases (GZMs), which are dependent on MHCII‐restricted antigen recognition.^45^ Our animal experiments validated the increase of CD4^+^ CTLs in both the brain and periphery of mice, accompanied by elevated apoptosis‐related proteins, which implies the direct cytolytic ability of the CD4^+^ CTLs in RIBI. However, the target cells remain unclear. A series of studies have confirmed the capacity of CD4^+^ CTLs to directly damage neurons, oligodendrocytes, and the BBB, indicating the pathogenic roles of CD4^+^ CTLs in neurological diseases.^45^, ^46^ Moreover, an investigation suggests that Vascular endothelial growth factor A (VEGF‐A) enhances the cytotoxic effects of CD4^+^ CTLs through AKT/mTOR signaling.^47^ VEGF‐A is crucial for the vascular response in the brain after irradiation, and therapeutic strategies targeting VEGF‐A (bevacizumab) and its downstream pathways have become clinical options for treating RIBI. The CD4^+^ CTLs may be an accomplice in inducing a series of damage responses in brain tissues after radiation. However, CD4^+^ CTLs also play roles in defending the body from viral infection and tumors, and a recent study found that CD4^+^ CTLs killed senescent cells, which supports the protective effects of CD4^+^ CTLs in some contexts.^29^ RIBI is a disease tightly associated with aging and tumors, so it is rational to consider that CD4^+^ CTLs might act as a protective component for the disease. The current observation of CD4^+^ CTLs in RIBI highlights their involvement in the immune responses related to RIBI pathogenesis, but the overall effect of CD4^+^ CTLs in RIBI, whether therapeutic or inflammatory, requires further investigation.

The CD4^+^ CTLs in RIBI also expressed high levels of *CCL5*, *CX3CR1*, and *CCL4L2* molecules. Our previous work has emphasized the significance of the CCL2/8‐CCR2/5 axis in the crosstalk between microglia and CD8^+^ cells promoting the RIBI.^20^ The abundance of chemokine and chemokine receptors on CD4^+^ CTL in RIBI further supports the close relationships between the immune systems and the CNS, which may be a key process in chronic inflammation of RIBI. CX3CR1 is a chemokine receptor commonly expressed in myeloid cells. Evidence suggests that CX3CR1 can be expressed in certain adaptive immune cells, reflecting their differentiation state and migratory properties.^48^ The CX3CR1 is a selective surface marker for CD4^+^ CTLs, allowing the distinction of this particular subcluster from other subpopulations in specific pathological conditions. Although the specific effects of CX3CR1 in CD4^+^ CTLs are unclear, a previous study in MS has demonstrated that CX3CR1 drives the migration of CD4^+^ CTLs into the CNS and enhances their cytotoxic activity.^44^ In the present study, high expression of *CX3CR1* corresponds to this subset being in a terminal differentiation state as identified by pseudotime analysis and provides insight into the generation and functional mechanisms of CD4^+^ CTLs in RIBI.

In addition to surface markers and secreted molecules, nuclear transcriptional regulators are crucial for determining the CD4^+^ CTL lineage. In this study, three TF‐encoding genes including *TBX21*, *RORB*, and *EOMES* were identified to distinguish the CD4^+^ CTL in RIBI. These genes are proven to induce the cytotoxic effects of CD4^+^ CTLs and have also been reported in other neurological diseases. However, the combination of TFs in RIBI seemed to be distinct from those in other neurological diseases as re‐analyzed results shown above. It may reflect the shared and distinct pathogenic mechanisms in these diseases. According to previous studies, the Eomes^+^ CD4^+^ CTL is capable of being a biomarker for SPMS diagnosis and prognosis with an accuracy of more than 80%.^27^ Therefore, exploring the relationships between CD4^+^ T cells and these TFs and the occurrence and development of disease may provide evidence for the potential of CD4^+^ CTLs as biomarkers for RIBI. Furthermore, targeting these TFs is hopeful to be an effective therapeutic strategy for RIBI treatment.

Coupled with the previous publication,^20^ our results here support that both CD8^+^ CTLs and CD4^+^ CTLs are linked to immune response in RIBI. Both lymphocytes induce apoptosis in their targeted cells through the secretion of cytotoxic granules at the molecular level. However, distinctions exist between the two. Firstly, as mentioned previously, CD4^+^ CTLs function in MHC class II‐dependent manner, while CD8^+^ CTLs target MHCI^+^ cells. Consequently, while CD8^+^ CTLs are confirmed to be pathogenic in the development of RIBI, whether CD4^+^ CTLs play a protective or pathogenic role in RIBI is to be explored, which may depend on the specific function of the cells they kill in the disease. The chemokine receptors and TFs prevalent in RIBI‐related CD4^+^ CTLs differ from those found in CD8^+^ CTLs, indicating potential variations in their differentiation routes and the recruitment into the brain. Furthermore, these phenotypic differences will also indicate that our intervention approaches for CD4^+^ CTLs in RIBI may be markedly distinct from those for CD8^+^ CTLs.

To our knowledge, it is the first study illustrating the single‐cell transcriptional landscape of CD4^+^ T cells and emphasizing the potential significance of CD4^+^ CTLs in RIBI. However, as a preliminary investigation, there are still limitations in the present study. Firstly, the sample size was relatively small, so the conclusion needs to be validated with future studies in larger cohorts. Secondly, there are limitations in displaying the CD4^+^ CTLs in vivo with conventional tools, because CD4^+^ CTLs represent a relatively small cell population in the brain, and GZMB, as a secreted protein, is also challenging to detect in situ. Therefore, further attempts using GZMB‐Tom‐KI transgenic mice or fluorescent probes may help visualize the cytotoxic granules and track the CD4^+^ CTLs in RIBI.^49^ Moreover, it is also necessary to look for suitable cell markers that are more specific and easier to detect for distinguishing the CD4^+^ CTL cluster. Thirdly, our present study focused solely on the CD4^+^ CTLs. It is imperative to validate other CD4 subclusters by animal or clinical experiments and to explore their roles in RIBI. Lastly, the specific roles and underlying mechanisms of CD4^+^ CTLs in the development of RIBI remain unclear. Interventions on this subset are of great significance in future studies.

## 5 CONCLUSION

Although it has some limitations, it is the first study to illustrate the transcriptional signatures and potential differentiation routes of CD4^+^ T cells in RIBI. A specific CD4^+^ CTL subset with cytotoxic capacities was clonally expanded in CNS, presenting with a terminal differentiation state and several genes as its key TFs. Our results shed light on the roles of adaptive immune response in the pathogenesis of RIBI, and CD4^+^ CTLs may serve as potential biomarkers or therapeutic targets for the disease.

## AUTHOR CONTRIBUTIONS

YMT, MJZ, and ZSS contributed to the conception and design of the study. XYM, YZ, and XH designed the experiments. XYM, RQX, KZ, KJL, JWY, XQZ, and SSG performed the experiments. YZ and STC contributed to the scRNA‐seq and TCR‐seq analyses. XYM and YZ drafted the manuscript. SJL, ZSS, XH, ZHD, XLL, and YTX revised the manuscript. All authors have participated in the review and editing.

## FUNDING INFORMATION

This work was supported by grants from the National Natural Science Foundation of China (Nos. 81925031 and 81820108026), Science and Technology Program of Guangzhou, China (202007030001), and STI 2030‐ Major Projects (2022ZD0211600) to Yamei Tang; National Natural Science Foundation of China (No. 82103775) and China Postdoctoral Science Foundation (2023T160756 and 2022M723589) to Zhongshan Shi; and China Postdoctoral Science Foundation (2023M731538) to Xia Hu.

## CONFLICT OF INTEREST STATEMENT

The authors declare no conflicts of interest.

## CONSENT TO PARTICIPATE

The patients/participants provided their written informed consent to participate in this study.

## CONSENT TO PUBLISH

The patients/participants provided their written informed consent to this publication.

## Supporting information


Figures S1–S3

## Data Availability

The original data in the current study are available from the corresponding author upon reasonable request.
