# Supplementary material for: Terminally differentiated cytotoxic CD4 + T cells were clonally expanded in the brain lesion of radiation‐induced brain injury
Source: CNS Neurosci Ther. 2024 Mar 18;30(3):e14682. doi: 10.1111/cns.14682 (PMC10948588; doi:10.1111/cns.14682)
Supplement: Supplementary file 1 — Figures S1–S3 [file CNS-30-e14682-s001.docx]

**Supplementary Information**

**Supplementary Figures**


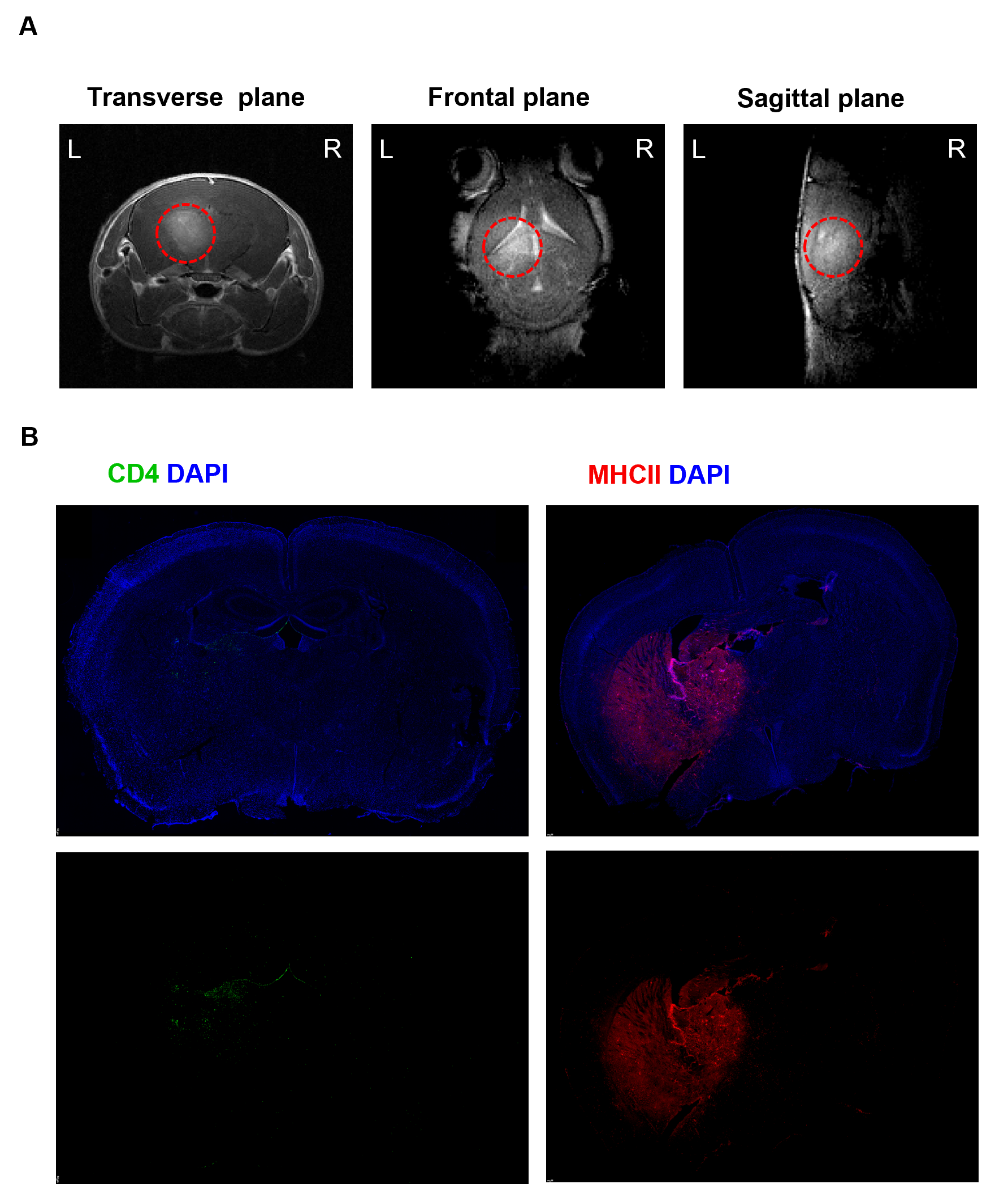


**Fig.S1 MRI and immunofluorescence (CD4 and MHCII staining) imaging of brain lesion of gamma knife irradiation mice**

1. Magnetic resonance imaging (T1WI sequencing) showed the brain lesion of gamma-knife irradiation mice at 8w after irradiation.
2. Immunofluorescence staining detecting CD4 (green) cells and MHCII (red) respectively in mouse brain after gamma knife irradiation.


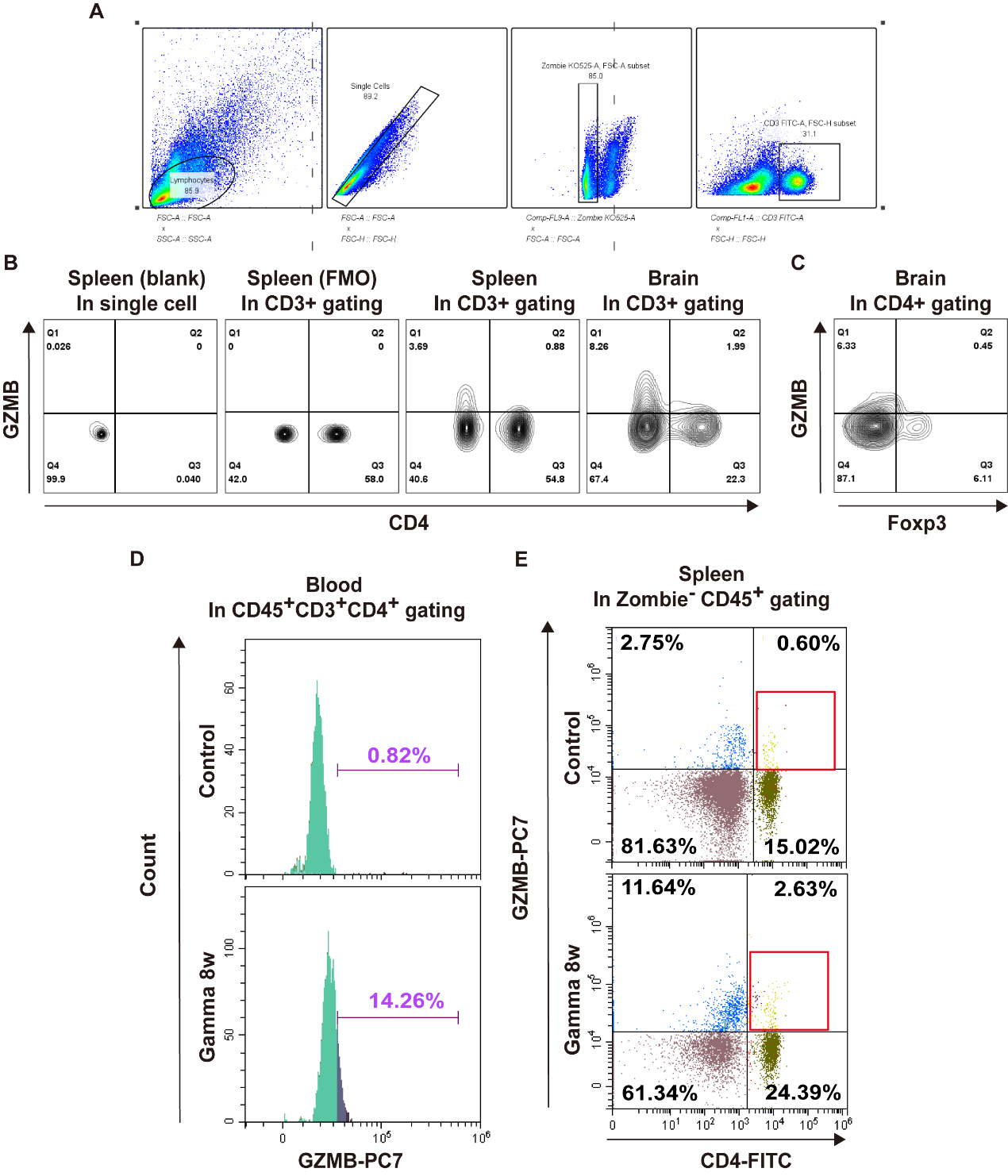


**Fig.S2 GZMB producing-CD4^+^ CTLs were increased in the periphery of gamma knife irradiation mice.**

1. Backgating strategy for (B) in splenocyte.
2. Scatter plots showing the expression of GZMB in both CD3^+^CD4^-^(mainly CD8+T cells) and CD4^+^T cells in blank (unstained sample), GZMB FMO sample, spleen, and brain tissue (from gamma knife irradiated mouse).
3. Foxp3 and GZMB expression in CD4^+^T cells from brain tissue of gamma knife-irradiated mouse.
4. Histogram gating the CD4^+^GZMB^+^ cells within the CD45^+^CD3^+^CD4^+^ population in the peripheral blood from gamma knife-irradiated mouse.
5. Scatter plots displaying the CD4^+^GZMB^+^ cells within the CD45^+^ gating in the splenocyte of gamma knife-irradiated mouse.


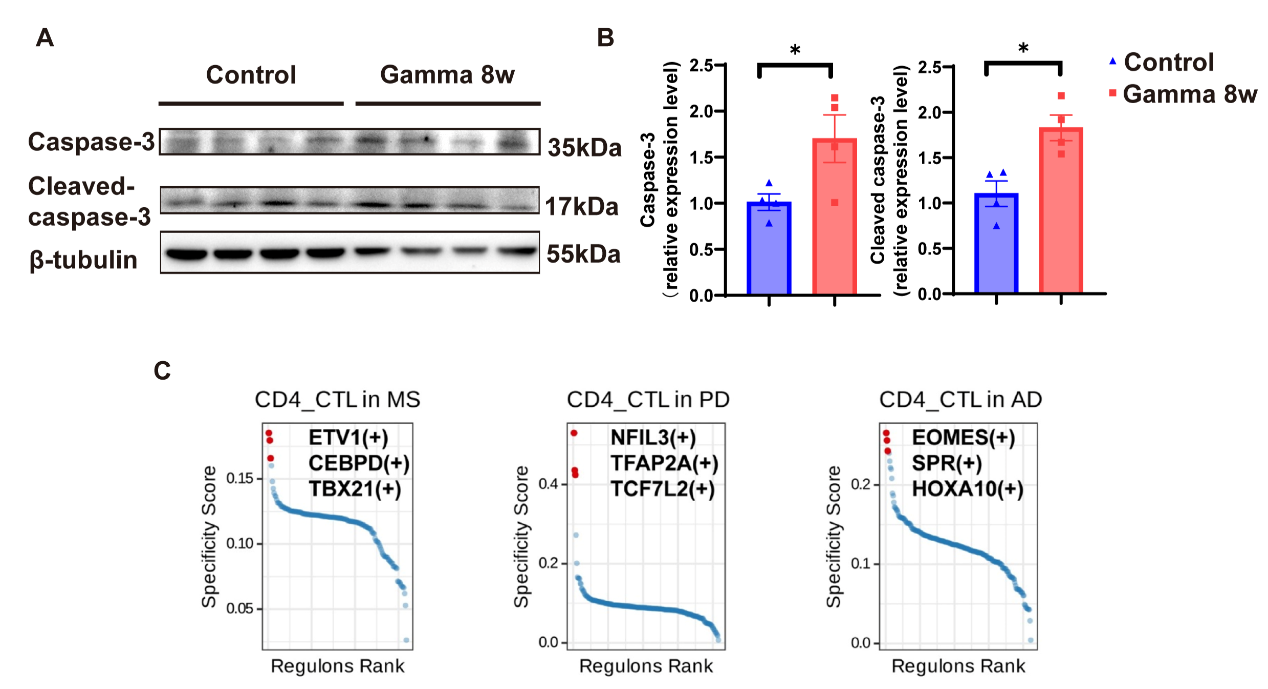


**Fig. S3** **Apoptosis-related proteins were increased in the periphery of gamma knife irradiation mice and key TFs for CD4^+^ CTLs in neurological diseases**

1. Western blotting analysis of caspase-3 and cleaved-caspase-3 protein expression level in the ipsilateral thalamus of healthy control and gamma knife-irradiation mice.
2. Quantification of protein expression level in the (A) and comparisons between the two groups.

Data were presented as mean ±SEM, and every point represented results from an individual. All the data passed the Shapiro–Wilk normality test, and unpaired two-tailed Student's t-tests

was utilized for two group comparisons (*p<0.05).

1. The scatter plot shows the specific regulons in CD4^+^ CTL of multiple sclerosis (MS), Parkinson’s disease (PD) and Alzheimer’s disease (AD), highlighting the top 3 regulons. The data were from published articles and public databases[1-3].

1. Ostkamp P, Deffner M, Schulte-Mecklenbeck A et al. A single-cell analysis framework allows for characterization of CSF leukocytes and their tissue of origin in multiple sclerosis. Sci Transl Med.2022; 14: eadc9778.

2. Wang P, Yao L, Luo M et al. Single-cell transcriptome and TCR profiling reveal activated and expanded T cell populations in Parkinson's disease. Cell Discov.2021; 7: 52.

3. Xu H, Jia J. Single-Cell RNA Sequencing of Peripheral Blood Reveals Immune Cell Signatures in Alzheimer's Disease. Front Immunol.2021; 12: 645666.
